# Coupled Effects of Polyethylene Microplastics and Cadmium on Soil–Plant Systems: Impact on Soil Properties and Cadmium Uptake in Lettuce

**DOI:** 10.3390/toxics13070555

**Published:** 2025-06-30

**Authors:** Zhiqin Zhang, Boyuan Bi

**Affiliations:** 1 School of Materials Engineering, Shanxi College of Technology, Shuozhou 036000, China; 2 College of Natural Resources and Environment, Northwest A&F University, Yangling, Xianyang 712100, China; 3 Shaanxi Key Laboratory of Qinling Ecological Intelligent Monitoring and Protection, School of Ecology and Environment, Northwestern Polytechnical University, Xi’an 710012, China

**Keywords:** microplastics, lettuces, combined pollution, soil–plant system, Cd uptake mechanism

## Abstract

Microplastics (MPs) and cadmium (Cd) in the soil environment are expected to pose a serious threat to agricultural production. However, the effect of the interaction between them on the soil–plant system and the mechanism of MPs on plant Cd uptake are still unclear. Therefore, the effects of different concentrations of polyethylene (PE-MPs, 0, 1.0% and 2.0%), alone or combined with Cd, on soil properties, plant growth and Cd uptake were investigated through pot experiments. The results showed that the single contamination of MPs and Cd and their interaction (MPs + Cd) significantly decreased soil moisture and pH; however, it increased soil organic matter (SOM) and total nitrogen (TN). Soil urease and catalase activities were significantly decreased and sucrase and alkaline phosphatase activities were increased with or without Cd addition. The exposure of PE and Cd, alone or combined, significantly and negatively affected plant biomass, photosynthetic parameters, and caused oxidative damage to plants, and the overall toxicity to plants increases with the increase in PE concentration. Moreover, co-pollution causes greater plant toxicity than the individual pollution of PE or Cd. Plants can resist oxidative stress by increasing superoxide dismutase (SOD) and peroxidase (POD) activities. The heat map showed that soil environmental factors were significantly correlated with plant growth; and the results of redundancy analysis (RDA) indicated that for plant physiological characteristics, soil properties under PE, alone or co-contaminated with Cd, explained a total of 85.77% and 97.45%, respectively. This indicated that the alteration of the soil microenvironment is the key factor influencing plant growth. The results of the partial least squares path model (PLS-PM) indicated that plant oxidative damage and biomass had significant positive and negative direct effects on plant Cd uptake, respectively. The linear model of relative importance (%) further revealed in depth that soil moisture (relative importance: 33.60%) and plant biomass (relative importance: 20.23%) were, respectively, regarded as the most important soil environmental factors and plant indicators affecting their Cd uptake. This study provided theoretical support for assessing the risks of MPs and Cd co-pollution to agricultural ecosystems.

## 1. Introduction

It is well known that plastics, as one of the greatest inventions of humankind, have brought great convenience to our lives while causing serious environmental pollution [1,2]. Currently, it is widely believed that soil is the ultimate sink for waste plastics [3]. Studies have demonstrated that more than 30% of the world’s waste plastics eventually enter the soil or agro-ecosystems, and large amounts of plastics are further decomposed into microplastics (MPs) with particle sizes smaller than 5 mm through physical, chemical and biological processes in the soil environment [4]. It is expected that MPs will persist in the soil for a long time and pose a potential threat to agricultural production by destroying soil structure and hindering crop growth [5].

Numerous studies have suggested that, unlike soil particles, the large accumulation of MPs in soil will inevitably have an impact on the structure and function of soil [2,6]. For example, MPs can be embedded in the microstructure of soil to varying degrees, hindering the effective integration of microaggregates with macroaggregates [7]. Meanwhile, MPs can alter soil permeability and water retention, and as well as soil porosity and bulk density, thereby affecting water evaporation [8]. Furthermore, MPs can have a general effect on soil nutrients and soil biochemical properties due to their different sizes, compositions, types, and other characteristics [2]. For example, MPs are synthetic polymers with high carbon content. MPs entering the soil may be disguised as an important anthropogenic component of the organic carbon pool [9], thereby exacerbating the C:N imbalance [10], and, further, they can have adverse effects on the community in the rhizosphere of plants [11]. In addition, MPs can significantly affect the structure of soil microbial communities. It is reported that MPs can serve as a new substrate for microbial colonization, forming a unique environment in the “plastic sphere” [12]. This stimulates the changes of specific groups of soil microorganisms and affects the interaction between plants and microorganisms [13]. In addition, soil enzymes play a crucial role in regulating soil nutrient cycling as indicators for assessing soil fertility [1]. Existing studies have widely demonstrated that the addition of MPs can significantly affect soil enzyme activities, mainly including urease, catalase, phosphatase and sucrase, etc. [14,15]. However, it is worth noting that the effects of MPs on soil nutrient cycling and enzyme activity are contradictory, possibly promoting, inhibiting or having no significant effect [16,17]. Therefore, paying attention to the impact of MPs on soil functions is conducive to further quantifying its environmental risks and ecotoxicity.

As an emerging pollutant in the soil environment, MPs not only affect soil health and function, but also lead to complex changes in the environmental behavior of various soil contaminants, including heavy metals, and further affect the soil–plant system [2,18]. Cadmium (Cd) is one of the major heavy metal pollutants in agricultural soils. Cd has been of great concern due to its persistence, high toxicity, easy migration and transformation, and ability to be readily absorbed by plants and endanger human health through the food chain [19,20]. MPs has a large specific surface area and complex surface characteristics and can become a favorable carrier of Cd through interactions such as adsorption [21,22]. The combination of MPs and Cd can change soil structure, fertility, soil microorganisms and enzyme activities, thereby having an impact on plants [23]. For instance, Wang et al. [24] showed that PE can induce synergistic toxicity in plant growth by altering the soil microenvironment, which can increase the bioavailability of soil Cd and lead to an increase in plant Cd concentration. In fact, the contamination of MPs with Cd may produce more serious hazards than a MPs contamination alone. This is because MPs reduce the ability of soil to adsorb heavy metals, leading to increased mobility of these metals in soil [19]. For example, it has been reported that the presence of PE-MPs (2.5% *w*/*w*) increased the availability of Cd and Zn in the soil and increased the concentration of heavy metals in lettuce leaves, corn shoots and roots [19,25]. However, it is worth noting that there is no uniform conclusion on the combined effect of MPs and Cd. It has been suggested that PE microplastics have no effect on the accumulation of heavy metal Cd in maize [26]. Some studies have also shown that PE-MPs can mitigate Cd stress in cabbage via soil–microbe–nutrient and Cd accumulation, regulation, etc., [27,28]. In summary, the combined effects of MPs and Cd on the soil–plant system are complex and diverse. It mainly depends on the type, quantity, and particle size of MPs and the exposure concentration of Cd, etc. [29]. Therefore, studying the interactions between MPs and Cd is necessary to accurately assess the risk of their combined effects in the soil–plant system.

Currently, our understanding of how MPs regulate the transport process and toxicity of Cd in the soil–plant system is still limited. Based on this, in this study PE and Cd were used as pollution factors through pot experiments, and the ecological effects and environmental behaviors of different concentrations of PE alone or combined with Cd on the soil–plant system were explored. The main objectives of this study were as follows: (1) To clarify the influence of the MPs-Cd pollution system on soil properties and enzyme activities; (2) To explore the impact of combined pollution on the physiological characteristics of plants; (3) To reveal the driving factors and regulatory mechanisms of MPs on Cd absorption in plants.

## 2. Materials and Methods

### 2.1. Experimental Materials Preparation

Before the experiment began, the MPs particles were pre-treated to remove the influence of impurities. The MPs used in this experiment were polyethylene (PE-MPs), with an average size of 75 μm (pellets), which was purchased from Taobao Huachuang Plastic Chemical Company. Before the experiment began, the MPs particles were pre-treated to remove the influence of impurities. Specifically, they were ultrasonically cleaned with 5% HNO_3_ two times, and then with deionized water two times. After cleaning, the MPs were naturally dried in the dark and stored in self-sealing bags for future use.

The test soil for this study was collected from the fresh surface soil (0–20 cm) of farmland in Yan’an City, Shaanxi Province, China (36°39′00″ N, 109°29′38″ E), and the soil samples were mixed well to homogenize the soil samples after multipoint sampling. Then, stones, plant roots, insects, plastic products or film impurities, etc., were immediately removed. Soil samples were stored in aluminum boxes and transported back to the laboratory to air dry naturally and then were sieved through a 2 mm sieve for later use.

In this study, Cd was introduced as a Cd solution (CdCl_2_·2.5H_2_O) into the soil–PE mixture, and CdCl_2_·2.5H_2_O was purchased from Shanghai, China, Shanghai Macklin Biochemical Co., Ltd. 

Lettuce seeds were purchased from Shouguang City, Shandong Province, China, Shouguang Xinxinran Horticulture Co., LTD, categorized as year-round lettuce. Before use, full-grained lettuce seeds were sterilized in 3% H_2_O_2_ for 30 min and thoroughly rinsed with distilled water to remove residue. Then, the seeds were soaked in distilled water for 12 h. The water on the seed surface was wiped off and used immediately.

### 2.2. Experimental Design and Procedure

Pot experiments were conducted using the pre-treated lettuce seeds and MPs mentioned above. A total of six treatments were set up in this experiment: CK (no MPs and Cd added, control treatment); PE1 (MPs 1.0%); PE2 (MPs 2.0%); Cd-only treatment (Cd); PEH1 (MPs 1.0% + Cd); and PEH2 (MPs 2.0% + Cd), and each treatment was replicated six times. The concentration settings of this experiment were referred to from a previous study [28,30].

The experimental procedure was as follows: first, five holes with a diameter of 1.2 cm were punched at the bottom of the pots to facilitate drainage, and then the bottom was covered with a piece of filter paper with a diameter of 15 mm to prevent the loss of soil and MPs. Before adding PE, the PE pellets were sterilized under UV irradiation for 30 min to reduce microbial contamination. Then, 1.8 kg of soil was mixed with the corresponding proportion of PE or Cd, and all materials were mixed well, and the soil was stabilized in equilibrium at room temperature for 30 days. Plant 10 uniformly sized, plump and disinfected seeds in each pot. The diameters of the top and bottom of the pot were 18 cm and 15 cm, respectively, and the height was 16 cm. After 7 days of growth, numbers were reduced to five seeds per pot. During the growth period, the soil moisture content was maintained at about 20% by the weighing method. The plants were harvested after being exposed to light for 12 h every day and growing for 40 days. Meanwhile, soil samples were collected for subsequent analysis and determination.

### 2.3. Determination and Analysis of Plant and Soil Indexes

#### 2.3.1. Plant Indexes

Determination of plant biomass: after harvesting, the plants were washed with deionized water, divided into shoots and roots, dried of excess deionized water with toilet paper, and then measured for fresh weight. The fresh or dry biomass of the plants was weighed by the weight of all plants in each pot (g pot^−1^). The plants were killed at 105 °C for 2 h and then dried at 60 °C for 48 h to a constant weight, and the fresh weights of stems and roots were determined separately.

Measurement of plant photosynthesis parameters: Photosynthesis parameters of plants were measured with a portable infrared gas analyzer (LI-6400, LI-COR Corporation in Lincoln, NE, USA) from 10:00 to 11:30 a.m. on the day before harvest. Five fully expanded second or third functional leaves were randomly selected from each treatment, and photosynthetic rate (Pn), stomatal conductance (Gs), instantaneous transpiration rate (Tr), and intercellular CO_2_ concentration (Ci) were measured.

Determination of plant oxidative damage and antioxidant enzyme activity: the plant oxidative damage indicators and antioxidant enzyme activity were determined using a kit (purchased from Suzhou Keming Biochemical Reagent Co., LTD.), mainly including hydrogen peroxide (H_2_O_2_), superoxide anion (O_2_·^−^), malondialdehyde content, as well as the activities of superoxide dismutase (SOD) and peroxidase (POD). Specifically, mature leaves from the same part were taken to measure physiological indicators. An amount of 0.3 g of plant leaf tissue was weighed, subsequently added to the extract, ground into a powder using liquid nitrogen, centrifuged and then measured using a UV spectrophotometer at a specific wavelength.

Determination of Cd in plants: The dried plant samples were ground to 4 mm in an agate mortar and then digested for the determination of heavy metals. Specifically, 0.500 g of the plant sample was weighed into a digestion tube and 10 mL of mixed acid (HNO_3_: HClO_4_ = 4:1) was added, digested completely and filtered. The filtrate was tested for heavy metals by flame atomic absorption photometer.

In addition, the uptake and transfer coefficients (TF) of heavy metals by plants were calculated by the following equations:

Plant total Cd uptake = shoot/root biomass × shoot/root Cd concentration

TF = metal content of stems and leaves/metal content of roots

#### 2.3.2. Soil Indexes

Determination of soil physical and chemical properties: Soil water content was determined by the “constant weight drying method” [31]. The soil pH value was measured in a suspension with a soil–water ratio of 1:2.5 (*w*/*v*) by using a pH meter with a glass electrode (InsMarkTM IS126, Shanghai, China). Soil organic carbon (SOM) was determined by the dichromate oxidation method [32]. Total nitrogen (TN) was determined by the Kjeldahl method [33]. Soil total phosphorus (TP) was determined by the molybdenum blue method [31].

Soil enzyme activities were determined by referring to the method of Duan et al. [34]. In this study, fresh soil was used for the determination of soil enzyme activities. Briefly, urease was determined by the sodium phenol-sodium hypochlorite colorimetric method and the enzyme activity was expressed as NH_3_-N mg; sucrase was determined by the 3,5-dinitrosalicylic acid colorimetric method and the enzyme activity was expressed as glucose content. Alkaline phosphatase was determined by the disodium phenylphosphate colorimetric method and the enzyme activity was expressed as phenol content. Catalase was determined by the potassium permanganate titration method and the enzyme activity was expressed as the residual amount of hydrogen peroxide after titration.

Determination of the total concentration of Cd in soil: Specifically, approximately 0.3 g of soil sample was weighed, 4.5 mL of HCl and 1.5 mL of HNO_3_ were added, and the sample was soaked overnight. After microwave digestion for 1 h, 0.5 mL of HClO_4_ was added and the acid was removed with a constant temperature digestion instrument for 2 h. The volume was made up, filtered, and stored. The concentration of Cd in the sample was determined by an atomic absorption spectrophotometer (Hitachi, Ltd, FAAS Z-2000, Tokyo, Japan). The available Cd was extracted using DTPA solution (0.005 M DTPA, 0.1 M triethanolamine, 0.01 M CaCl_2_, pH = 7.3). The suspension (soil: DTPA solution ratio, 1:2, *w*/*v*) was shaken at 180 rpm at 25 °C for 2 h, and then filtered with filter paper and a Millipore 45 μm filter. Finally, the Cd concentration was determined via atomic absorption spectrophotometry (Hitachi, FAAS Z-2000, Japan).

### 2.4. Statistical Analysis

Statistical analyses were performed using IBM SPSS Statistics 27. A one-way ANOVA was used to compare the differences between the indicators under different treatments, and a two-way ANOVA was used to determine the main and interaction effects of MPs concentration and whether or not Cd was added on each indicator. Data in graphs and tables were presented as mean ± standard deviation. All data were generated using Origin 2025. Correlation heat maps between plant growth indicators and soil properties (as well as heavy metal indicators) were plotted using the R software packages “ggcorrplot” and “linkET”. Redundancy (RDA) analysis of plant physiological indicators and soil environmental factors was performed using Canoco5 (Version 5.0). In addition, we used partial least squares path modeling (PLS-PM) to reveal possible pathways of predictor variables controlling plant Cd uptake. Finally, the relative importance of soil environmental factors and plant physiological indicators as drivers of Cd uptake was analyzed using the R package (Version 4.2.2) “random forest”.

## 3. Results

### 3.1. Soil Physicochemical Properties and Enzyme Activities

The pollution of MPs alone and with Cd and their interaction (MPs + Cd) significantly (*p* < 0.05) affected the physical and chemical properties of the soil, including soil moisture, pH, SOM, TN and TP (Table S1). Specifically, regardless of whether Cd was added or not, compared with CK treatment, PE exposure significantly reduced soil moisture and pH, while stimulating SOM content. In addition, PE addition led to an increase in soil TN content and reached a maximum of 0.21 g kg^−1^ under the PE2 treatment. TP content was significantly higher under PE exposure alone than co-contamination; however, there was little difference among the treatments regardless of whether Cd was added or not. Overall, the results of two-way ANOVA demonstrated that the PE concentration had a significant main effect on almost all soil physicochemical properties, except for TP. Cd had significant main effects on moisture, pH and TP. However, MPs and Cd had significant interaction effects on moisture, SOM and TN (Table S2).

**Figure 1:**
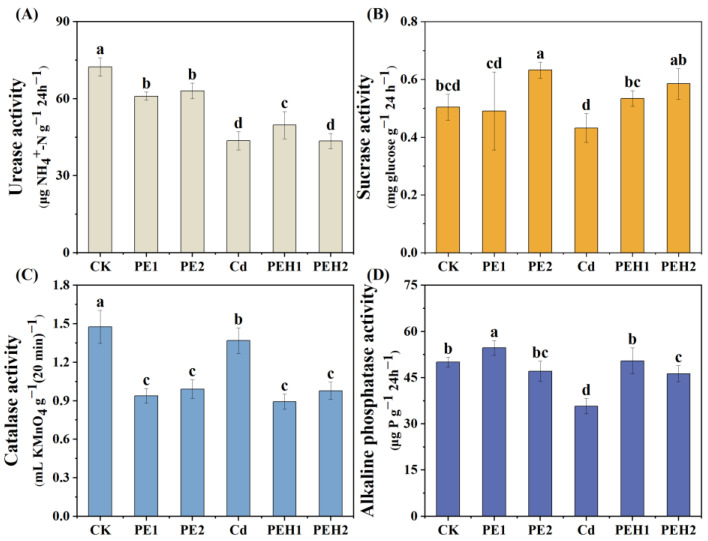
The combined effect of MPs and cadmium on the enzyme activities of soil. (**A**) statistical data of urease activity. (**B**) statistical data of sucrase activity. (**C**) statistical data of root catalase activity. (**D**) statistical data of alkaline phosphatase activity. Note: Data are means ± SD (*n* = 3). Different letters indicate a significant difference in the results; the same letters indicate a non-significant difference in the results (*p* < 0.05). CK: Control, PE1: 1.0% PE, PE2: 2.0% PE, PEH1: Cd + 1.0% PE, PEH2: Cd + 2.0% PE.

The activities of sucrase, alkaline phosphatase, urease, and catalase were affected by MPs exposure either alone or in combination with Cd (Figure 1). In general, compared with the CK treatment, soil urease and catalase activities were significantly reduced under the single addition of PE or combined exposure with Cd (*p* < 0.01), with co-contamination exerting a stronger inhibitory effect on enzyme activity (Figure 1A,C). Moreover, PE addition led to a significant increase in soil sucrase activity, whether or not co-pollution occurred, generally exhibiting a concentration–dose relationship. However, sucrase activity under PE + Cd treatment was consistently lower than that observed with PE added alone (Figure 1B). Compared with the CK treatment, soil alkaline phosphatase activity increased under the 1.0%-PE treatment but decreased significantly in other treatments. The alkaline phosphatase activity was the lowest under the treatment of Cd alone, and the enzyme activity of combined contamination was lower than that of MPs alone (Figure 1D). Similarly, the results of two-way ANOVA (Table S2) indicated that PE concentration had a significant main effect on the activities of the four enzymes in the soil (*p* < 0.05), and that PE and Cd exhibited a significant interaction effect on soil urease and alkaline phosphatase (*p* < 0.001).

### 3.2. Plant Biomass and Photosynthesis Parameters

**Figure 2:**
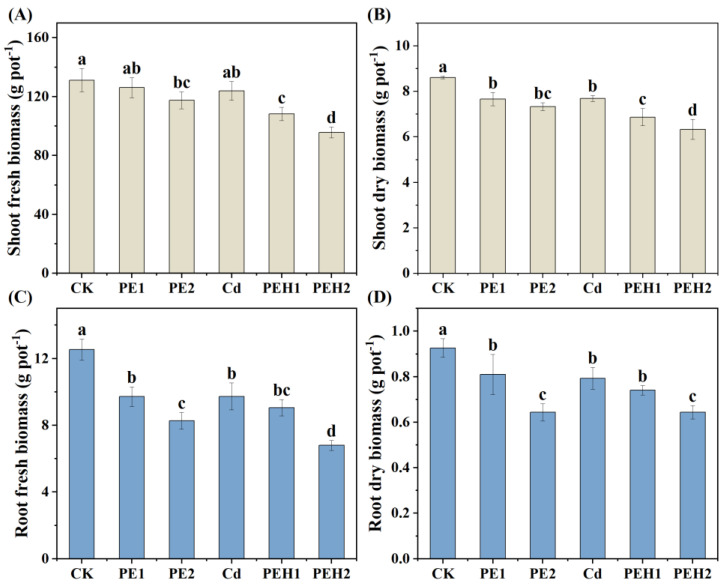
The combined effect of MPs and Cd on the biomass of plant shoot and root. (**A**) statistical data of shoot fresh biomass. (**B**) statistical data of shoot dry biomass. (**C**) statistical data of root fresh biomass. (**D**) statistical data of root dry biomass. Note: Data are means ± SD (*n* = 3). Different letters indicate a significant difference in the results; the same letters indicate a non-significant difference in the results (*p* < 0.05). CK: Control, PE1: 1.0% PE, PE2: 2.0% PE, PEH1: Cd + 1.0% PE, PEH2: Cd + 2.0% PE.

The biomass of lettuce shoots and roots under different treatments was presented in Figure 2. The results showed that MPs treatment alone significantly reduced the fresh and dry weight of lettuce (*p* < 0.01), and the degree of inhibition increased with the increase in PE concentration. For example, compared with the CK treatment, under the PE1 and PE2 treatments, the dry weights of lettuce shoots and roots decreased by 11% and 13%, as well as 15% and 31%, respectively. Furthermore, compared with the single PE treatment, the PE + Cd treatment further reduced the biomass of lettuce. In particular, the dry weight of shoots had a significant concentration–dose effect on MPs. Furthermore, the results of the two-way ANOVA (Table S2) showed that PE and Cd had significant main effects on both the shoots and roots of fresh or dry biomass, while they had significant interaction effects on root fresh biomass (*p* < 0.5).

The photosynthesis parameters were measured and shown in Table 1. Specifically, under the CK treatment, lettuce had the largest Pn, Gs, and Tr values and the smallest Ci values. Ci was increased compared to the CK treatment. In addition, the photosynthesis of lettuce was further inhibited under the combined PE + Cd treatment compared to PE exposure alone. However, the degree of inhibition did not increase with increasing PE concentration. Furthermore, compared with the single Cd treatment, the Ci of the PE + Cd treatment increased significantly (*p* < 0.0.1) with the increase in PE concentration, while the Tr was the opposite. There was no significant difference in Pn between different treatments. Overall, the results of two-way ANOVA (Table S2) showed that PE and Cd had significant main and interaction effects on photosynthesis parameters in plants.

### 3.3. Plant Oxidative Damage and Antioxidant Enzyme Activity

**Figure 3:**
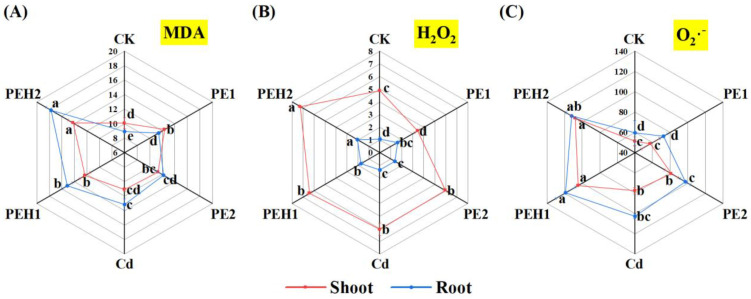
The combined effect of MPs and Cd on the oxidative damage to plants shoot and root. (**A**) statistical data of MDA content. (**B**) statistical data of H_2_O_2_ content. (**C**) statistical data of O_2_·^−^ content. Note: Data are means ± SD (*n* = 3). Different letters indicate a significant difference in the results; the same letters indicate a non-significant difference in the results (*p* < 0.05). CK: Control, PE1: 1.0% PE, PE2: 2.0% PE, PEH1: Cd + 1.0% PE, PEH2: Cd + 2.0% PE.

The contents of oxidative damage (MDA, H_2_O_2_ and O_2_·^−^) in plant tissues were shown in Figure 3. The results showed that, compared with the CK treatment, both PE single exposure or combined with Cd led to a significant increase in the content of oxidative damage in plant shoots and roots. In addition, co-contamination led to higher stress in plants compared to single PE pollution, and almost all indicators reached the maximum value under the treatment of 2.0%PE + Cd. Whether Cd was added or not, the content of MDA (Figure 3A) in shoots or roots had a significant concentration–dose effect with PE. Overall, the H_2_O_2_ content was significantly higher in shoots than in roots (Figure 3B), whereas O_2_·^−^ content (Figure 3C) was relatively higher in roots than in shoots. The two-way ANOVA showed that Cd had a significant main effect on all oxidative damage indicators of plant shoots and roots, and significant interaction effects on H_2_O_2_ and O_2_·^−^ (Table S2).

The SOD and POD activities of plant shoots and roots were presented in Figure S1A and S1B, respectively. Significant differences (*p* < 0.05) were observed in the activities of antioxidant enzymes under different treatments. Overall, the enzyme activities of plant shoots were basically significantly higher than those of roots, and the combined pollution of PE and Cd was significantly higher than the single pollution of PE. Specifically, plant antioxidant enzyme activities did not increase with PE concentration under PE addition alone, except for POD enzyme in shoots. However, the combined pollution led to a significant (*p* < 0.05) increase in the activities of all enzymes with the increase in PE concentration. Similarly, two-way ANOVA showed that both PE concentration and Cd had significant main effects on plant enzyme activities, as well as significant interaction effects on enzyme activities of roots (Table S2).

### 3.4. Cadmium Concentration in Plant and Soil

Table 2 demonstrated the effects of different concentrations of MPs added on Cd concentration in soil, plant shoots and roots. The results showed that the concentration of Cd in the roots of plants was significantly higher than that in the shoots. Furthermore, compared with the single Cd treatment, the addition of MPs further exacerbated Cd enrichment in plant tissues. Specifically, with the increase in PE concentration, the Cd concentration in the shoots showed a significant concentration–dose effect, and the Cd concentration in the roots also reached the maximum value under the treatment of 2.0%PE + Cd, which was 50.80 mg kg^−1^. In addition, TF and plant shoots Cd uptake were also maximum under 2.0%PE + Cd treatment, while the minimum Cd uptake in roots was due to the reduction in plant root dry biomass. Furthermore, soil total Cd concentration and DTPA-Cd concentration varied among treatments. Overall, PE addition increased the bioavailability of soil Cd. The results of two-way ANOVA (Table S2) showed that there were significant main and interaction effects (*p* < 0.01) of PE concentration and Cd on all indicators of Cd in plants and soil.

### 3.5. The Relationship Between Soil Environmental Factors and Plant Growth Characteristics

**Figure 4:**
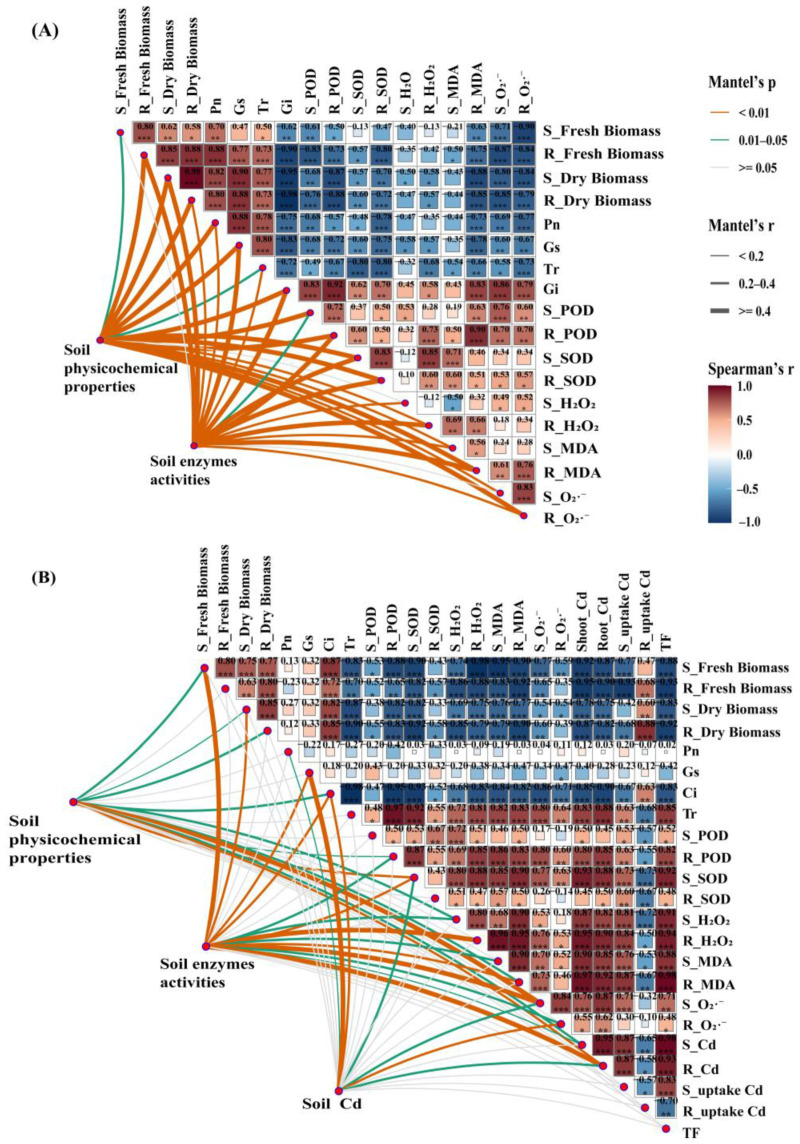
The correlation heat map of soil environmental factors and plant physiological indicators based on Mantel test. (**A**) Single exposure of MPs. (**B**) Combined exposure of MPs and Cd. Note: S: Shoot; R: Root. *, *p* < 0.05; **, *p* < 0.01; ***, *p* < 0.001.

The correlations between soil environmental factors and plant physiological properties under PE, alone or combined with Cd contamination, are presented in Figure 4A and Figure 4B, respectively. The results showed that only soil enzyme activities were not correlated with plant shoot fresh biomass and O_2_·^−^ under PE single contamination, while the rest of the soil indicators were significantly correlated (*p* < 0.05) with plant indicators (Figure 4A). For the combined PE and Cd pollution, the results suggested that soil physicochemical properties were significantly correlated with the shoots biomass, Tr, partial root oxidative damage, and plant Cd uptake. Soil enzyme activities had a weak correlation with root biomass, Pn, Gs and plant Cd uptake, while exhibiting a strong correlation with other indicators. The Cd in the soil was significantly correlated with photosynthetic parameters, SOD and O_2_·^−^ in shoots, and Cd uptake in roots, but not correlated with other indicators (Figure 4B). Furthermore, regardless of whether Cd was added or not, plant oxidative damage and antioxidant enzyme activity were generally significantly negatively correlated with plant biomass. Meanwhile, plant Cd concentration and TF were also significantly negatively correlated with their biomass and photosynthesis parameters, while significantly positively correlated with plant oxidative damage and antioxidant enzyme activities under combined PE and Cd pollution.

**Figure 5:**
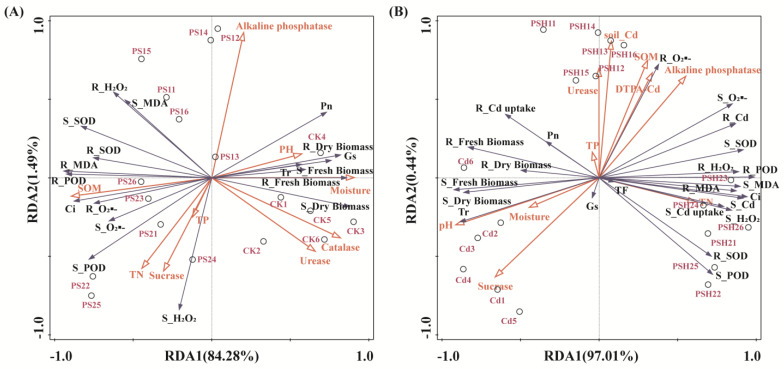
RDA analysis of plant physiological indicators and soil environmental factors. (**A**) Single exposure of MPs. (**B**) Combined exposure of MPs and Cd. Note: S: Shoot; R: Root.

To further understand the relationship between soil environmental factors and plant growth characteristics, RDA was conducted, as illustrated in Figure 5. The results showed that for PE single contamination, soil properties explained a total of 85.77%, and the first and second axes explained 84.28% and 1.49%, respectively. Specifically, soil moisture, pH and enzyme activities (except sucrase) were the main positive factors affecting plant biomass, while TN, TP and SOM were the main positive factors affecting plant oxidative damage and antioxidant enzyme activities (Figure 5A). For PE and Cd co-contamination, soil properties explained a total of 97.45%, while the first and second axes explained 97.01% and 0.44%, respectively. Specifically, soil pH, moisture, TP and sucrase were the main positive factors affecting plant biomass, while alkaline phosphatase and DTPA-Cd were the main positive factors affecting plant oxidative damage and Cd in plants (Figure 5B).

### 3.6. Factors Affecting Cd Uptake and Enrichment in Plants

**Figure 6:**
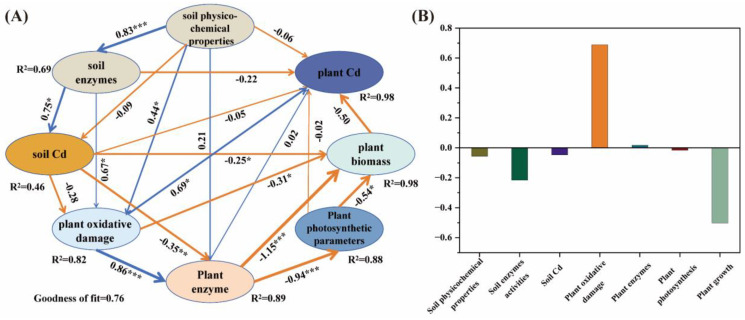
Partial least squares path modeling (PLS-PM) showed the direct and indirect effects of environment variables on Cd uptake in plant (**A**,**B**). Soil physicochemical properties: moisture, pH, SOM, TN. Soil enzymes: urease, alkaline phosphatase, catalase, sucrase enzymes. Soil Cd: soil total Cd, DTPA−Cd. Plant oxidative damage: H_2_O_2_, MDA and O_2_·^−^ in shoots and roots. Plant enzymes: POD and SOD enzymes in in shoots and roots. Plant photosynthetic parameters: Pn, Gs, Tr, Ci. Plant biomass: the fresh weight and dry weight of shoots and roots. Plant Cd: The Cd content and Cd uptake of shoots and roots. *, *p* < 0.05; **, *p* < 0.01; ***, *p* < 0.001.

PLS-PM was adopted to further evaluate the direct and indirect effects of soil physicochemical properties and plant physiological characteristics on plant Cd uptake (Figure 6A). The results showed that the direct effects of plant oxidative damage (0.67) and plant enzyme activities (0.02) on plant Cd uptake were positive. While soil physicochemical properties (−0.06), soil enzyme activities (−0.22), soil Cd concentration (−0.05), photosynthetic parameters (−0.02) and biomass (−0.50) were negative (Figure 6B).

Finally, in order to elucidate the effects of environmental variables on Cd uptake, a linear model of relative importance (%) was constructed using soil properties and plant physiological properties as environmental variables, respectively. The results showed that the model could explain 52.61% (Figure S2A) and 91.50% (Figure S2B) of the variation in Cd uptake by soil environmental factors and plant physiological characteristics, respectively. The results showed that moisture (relative importance: 33.60%) and fresh biomass (relative importance: 20.23%) were considered as the most important soil environmental factors and plant indicators affecting Cd uptake by plants, respectively.

## 4. Discussion

### 4.1. Effect of MPs Alone or Combined with Cd on Soil Properties

Currently, numerous studies have confirmed that the presence of MPs generally alters soil properties, such as soil bulk density, porosity, water-holding capacity, dissolved organic matter content (DOC), pH, and soil cation exchange capacity (CEC) [3,35,36]. Similarly, this study showed that compared to the control (CK), MPs (alone or combined with Cd) significantly reduced soil moisture, with PE-onlypollution causing progressively lower moisture at higher concentrations (*p* < 0.05). This might be due to the following reasons: Firstly, MPs are hydrophobic substances and have a rough surface, which affects water infiltration and evaporation, and thus has a greater effect on moisture with increasing PE concentration; secondly, as a type of solid pollutant, MPs will combine with soil particles after entering the soil and indirectly affect the soil moisture content by changing the soil structure, porosity and aggregates [35]. Finally, MPs tend to create cracks on the soil surface. They may disrupt the original structure of the soil surface and alter the properties of the interface between the soil and MPs, thereby reducing soil moisture [37]. In addition, this study showed that PE, alone or combined with Cd, decreased soil pH. Studies have indicated that the effect of MPs on soil pH depends on several factors, including the type and proportion of MPs and coexisting environmental factors, such as pollutants and planting period [38]. This may be due to, on the one hand, the aging degradation of MPs, during which compounds contained in MPs may be released, thus affecting soil pH [39]; and, secondly, changes in the soil microenvironment, root exudation, or microbial colonization of the surface of MPs leading to changes in soil pH [40].

MPs can significantly affect the nutrient cycle of the soil. The results of this study showed that the addition of PE led to an increase in soil SOM content (Table S1). On the one hand, it might be due to the increase in soil microbial activity that leads to the increase in SOM. Studies have suggested that MPs provide food and habitats for soil microorganisms [41]. Secondly, as a carbonaceous organic material, MPs can be detected by the current TOC quantification method [42], thereby increasing the content of SOM. Furthermore, the results of this study revealed that the addition of PE significantly increased the content of TN in the soil (Table S1). This is because MPs can significantly alter the microbial communities related to nitrogen transformation in the soil, resulting in changes in the TN content of the soil exposed to MPs. For example, Fei et al. [43] indicated that PVC significantly increased soil TN content after 22 days of incubation.

Enzymes can directly participate in the soil biogeochemical cycle. The results of this study showed that, on the whole, compared with the CK treatment, the addition of PE alone or the combined treatment with Cd significantly reduced soil urease and catalase (*p* < 0.01), and the co-contamination of PE and Cd had a stronger inhibitory effect on enzyme activity (Figure 1A,C). The results of Chen [44] et al. also demonstrated that the exposure of PLA-MPs significantly reduced the β-glucosidase urease and catalase activities. This is due to the fact that the addition of organic matter can stimulate the formation of soil aggregates, resulting in a large amount of MPs in soil aggregates, thereby reducing the stability of aggregates and further having a negative impact on enzyme activity [45]. The number and activity of microorganisms in the soil have an effect on catalase activity, and they are influenced to some extent by the physicochemical properties of the soil [46]. The decrease in catalase activity in this experiment reflects that the addition of MPs does not allow for the full decomposition of H_2_O_2_, resulting in a significant decrease in the ability of the soil to resist external toxicity, thus affecting soil metabolism [47]. In addition, Li et al. [40] found that urease activity was reduced in PE and heavy metal co-contaminated environments, which had implications for soil nitrogen cycling and microbial functioning. Liu et al. [48] indicated that the addition of MPs might affect the abundance of soil microorganisms involved in nitrogen transformation and influence the nitrogen cycle, thereby inhibiting urease activity. For soil alkaline phosphatase, overall, it was significantly reduced by PE addition and co-contamination led to further inhibition of enzyme activity (Figure 1D). A previous study indicated that phosphatase activity was negatively affected by increased SOM [49]. In addition, MPs can also adsorb essential nutrients such as phosphorus, reducing their availability and damaging the function of phosphatase. Finally, the presence of toxic substances, such as plasticizers and flame retardants, in MPs can further inhibit microbial enzyme activity and growth under Cd co-contamination. Furthermore, the addition of PE resulted in a significant increase in soil sucrase activity, essentially showing a concentration–dose effect (Figure 1B), the results of this study were similar to Li et al. [50]. The increase in sucrase may be related to the carbonization of soil SOM and the increase in SOC content caused by MPs as a carbon source. However, it has also been reported that the addition of PS or PE to soil resulted in a decrease in sucrase activity [51,52]. The differences in soil characteristics and polymer types may be the reasons for the variations in the results [3].

### 4.2. Effect of MPs Alone or Combined with Cd on Plant Growth

MPs have been widely proved to affect plant biomass [22,53]. The results of this study also showed that PE addition significantly reduced plant biomass with a significant concentration–dose effect (Figure 2). This may be attributed to the fact that PE tends to adhere to plant roots and hinders plants from obtaining nutrients. Furthermore, it has been revealed that the addition of MPs decreases soil bulk density and increases aeration [36]. Moreover, soil aeration may contribute to root penetration deeper into the soil and adversely affect plant development and growth [22,37]. Furthermore, PE combined with Cd contamination resulted in further inhibition of plant growth. Studies have demonstrated that the addition of MPs leads to a decrease in the adsorption capacity of heavy metals and an increase in the desorption capacity of metals in soil [24,54]. This means that the presence of MPs amplifies the bioavailability of soil Cd and the bioaccumulation of Cd [55].

The photosynthesis of plants is of great significance to their growth. The results of this study showed that MPs and Cd contamination significantly affected photosynthetic parameters of plants (Table 1). Specifically, compared with the CK treatment, PE exposure alone significantly inhibited the photosynthetic parameters of lettuce and increased Ci. In addition, the combined PE + Cd treatment inhibited plant photosynthesis more than PE alone. The results of this study were consistent with those of previous studies [40]. The decrease in photosynthesis in plants may be attributed to several factors. On the one hand, after plants were subjected to double stress, the effective pigments involved in photosynthesis decreased. The reaction center of Photosystem II was damaged due to exposure to MPs and Cd, and the photoenergy conversion efficiency decreased [56]. Secondly, the combined pollution might prompt PE to inhibit the carboxylation of ribosyl-1, 5-diphosphate in plants and intensify the effect of Cd, thereby inhibiting photosynthesis [57]. Furthermore, it has been demonstrated that MPs can affect root absorption capacity and ultimately have a negative impact on photosynthetic efficiency indirectly by deteriorating the overall performance of plants [58]. Finally, oxidative stress produced by plants can further affect their photosynthesis [28].

The results of this study showed that PE, alone or combined with Cd, increased the levels of oxidative damage in plants, and the co-pollution caused more intense oxidative stress to plants. This is due to oxidative stress on plant function caused by exposure to MPs and the interaction effect with Cd, and these results were consistent with previous studies [40,59]. It has been revealed that under PE adhesion or Cd stress, mechanical damage can be caused to the root surface or roots [60], leading to the production of ROS. However, plants protect their cells from excess reactive oxygen species by producing antioxidant enzymes [61]. The enzymes of POD and SOD play important roles in protecting against oxidative damage and eliminating superoxide radicals. SOD is the first line of defense for plants and is critical in the conversion of O_2_·^−^ to H_2_O_2_, whereas POD reduces the adverse effects of H_2_O_2_ on the plant [62]. Both correlation heatmap (Figure 5) and RDA analysis (Figure 6) showed that antioxidant enzyme activities in plants had a significant positive correlation with oxidative damage. In addition, there was no significant concentration–dose effect of antioxidant enzyme activity with increasing concentration of MPs under PE contamination alone. This is because when plants are exposed to low concentrations of MPs, in order to avoid external stress and toxic effects, plants induce an increase in the activity of antioxidant enzymes by promoting the operation of the antioxidant system, thereby reducing the accumulation of ROS. However, high concentrations of MPs can damage the antioxidant defense system and disrupt its balance [2].

### 4.3. Effect of MPs Alone or Combined with Cd on Plant Cd Uptake

Higher plants are an important component of terrestrial ecosystems and are widely used to detect and evaluate the toxicity of various environmental pollutants [63]. When emerging organic pollutants, such as MPs, enter soil ecosystems, they inevitably encounter metal pollutants and capture them onto negatively charged surfaces through π-π interactions, electrostatic interactions, and oxygen/hydrogen functional groups [64,65]. Recently, studies have investigated the influence of MPs on the bioavailability of Cd in the soil environment [22,26]. However, the relevant mechanisms and driving factors of Cd uptake in advanced plants under combined exposure with MPs are still lacking. The results of this study indicated that the presence of PE further stimulated the concentration of Cd in plant shoots and roots (Table 2), and the uptake of Cd by shoots under the treatment of 2.0%PE + Cd was significantly increased by 28.20% compared with single Cd exposure. This result was consistent with previous studies [29,51,66]. A meta-analysis displayed that MPs can enhance the bioavailability of numerous heavy metals such as Cd, Cu, Pb, and Mn [67]. And the significant increase in soil DTPA-Cd content under the addition of PE in this study also indirectly confirmed this point (Table 2). In addition, it is well known that soil pH significantly affects the morphology and bioavailability of Cd in soil–plant systems. The results of this study showed that addition of MPs decreased soil pH (Table S1). The negative charge of the soil solid surface is affected by pH, which alters the ability of Cd^2+^ ions to bind to the negative charge exchange sites on the solid surface [68].

However, the mechanisms affecting plant Cd uptake and enrichment under co-pollution of MPs and Cd may be quite complex [67]. For example, some studies suggest that the addition of MPs provides adsorption sites for Cd and may alleviate heavy metal stress [28,69]. Therefore, to further reveal the relevant mechanisms and driving factors influencing Cd uptake in plants, PLS-PM analysis (Figure 6) and the linear model of relative importance (%) analysis (Figure S2) were conducted in this study. The results showed that oxidative damage in plants had a significant positive effect (*p* < 0.01) on Cd concentration in their tissues and Cd uptake in shoots (Figure 6B). The correlation heat map (Figure 4B) and RDA analysis (Figure 5B) also confirmed this. It has been demonstrated that when plants are under stress, the excessive production of ROS can lead to cell membrane damage, thereby causing an increase in lipid peroxidation [70]. Meanwhile, oxidative damage indicators, especially O_2_·^−^ and MDA content, are the main indicators of plant response to Cd stress [71]. The linear model of relative importance (%) also indicated that O_2_·^−^ (12.02%) and H_2_O_2_ (11.91%) were considered very important variables affecting plant Cd uptake (Figure S2B). Since plant Cd uptake was directly calculated from biomass, it was considered the most important plant influence factor (Figure 6B and Figure S2B).

In addition, MPs can indirectly affect plant Cd uptake by changing soil properties and functions [22]. The PLS-PM results indicated that soil enzymes were important environmental factors affecting plant Cd uptake (Figure 6B). For example, catalase is one of the most important and sensitive bioindicators of heavy metal contaminated soils. Cd can affect the kinetic properties of catalase, thus limiting the catalytic reaction of the enzyme [55]. The addition of MPs will affect the soil sucrase activity by influencing soil microbial activity, while the combined pollution of MPs and Cd further alters the soil microbial community, thereby enhancing the bioavailability of Cd [51]. In addition, the random forest model showed that soil moisture was the most critical factor affecting plant heavy metal uptake (Figure S2A). It was found that plant uptake of nutrients/pollutants is closely related to soil moisture, whereas MPs addition can change the soil’s physical structure and directly affect soil water evaporation and water-holding capacity [8]. It is speculated that MPs can inhibit plants from absorbing water from the soil and transporting it upwards to the above-ground parts, thereby reducing the transportation of nutrients and pollutants [39]. In conclusion, the mechanism of MPs on plant Cd uptake and accumulation is relatively complex, and is affected by a variety of factors, such as the type and concentration of MPs, as well as soil conditions and plant species [29], which need to be investigated in depth in the future.

## 5. Conclusions

In this study, we conducted a 40-day pot experiment to reveal the effects of MPs alone or combined with Cd exposure on the soil–plant system, as well as to investigate the mechanisms and drivers of MPs addition on plant Cd uptake. Exposure to PE and Cd alone or in combination had a significant effect on soil properties. Specifically, these effects manifested as a reduction in soil moisture and pH, while increasing the contents of SOM and TN. The content of soil TP was higher under the single pollution of PE than the combined pollution of PE + Cd, but the differences between treatments were not significant. Compared with CK treatment, the addition of PE led to a significant decrease in the activities of soil urease and catalase, and an increase in the activities of sucrase and alkaline phosphatase. However, the enzyme activity under the combined pollution treatment was significantly lower than that under the single PE treatment.

Exposure to PE and Cd alone or in combination had a significant effect on plant growth. Specifically, the exposure of PE and Cd alone or combined had significant negative effects on plant biomass and photosynthetic parameters, caused oxidative damage to plants, and increased overall toxicity to plants with increasing PE concentration. Plants can resist oxidative stress caused by exogenous stress by increasing the activities of SOD and POD. The heat map showed that soil environmental factors were significantly correlated with plant growth. Moreover, the results of redundancy analysis (RDA) indicated that for the physiological characteristics of plants, the soil properties under PE alone or combined with Cd pollution explained a total of 85.77% and 97.45%, respectively. This indicates that the alteration of the soil microenvironment is the key factor influencing plant growth.

Furthermore, in this study, PLS-PM was constructed to explore the influencing mechanism of Cd absorption in plants, and the linear model of relative importance (%) further revealed the driving factors of Cd uptake in plants. The results showed that the direct effects of plant oxidative damage (0.67) and antioxidant enzymes (0.02) on the uptake of Cd in plants were both positive, while the other indicators were negative. The results of the linear model of relative importance exhibited that the model could explain 52.61% of soil environmental factors and 91.50% of plant physiological characteristics on the variation of plant Cd uptake, respectively. And moisture (relative effect: 33.60%) and plant biomass (relative effect: 20.23%) were considered as the most important soil environmental factors and plant indicators, respectively.

## Figures and Tables

**Table 1:** The combined effect of MPs and cadmium on the photosynthetic parameters of plant.

Treatments	Pn	Gs	Tr	Ci
	(μmol CO_2_ m^−2^ s^−1^)	(mol H_2_O m^−2^ s^−1^)	(Mmol H_2_O m^−2^ s^−1^)	(μmol CO_2_ mol^−1^)
CK	16.86 ± 0.32 a	0.31 ± 0.02 a	11.70 ± 0.89 a	204.01 ± 1.89 d
PE1	16.56 ± 0.09 ab	0.24 ± 0.04 bc	10.75 ± 0.30 ab	222.32 ± 0.50 c
PE2	15.57 ± 0.25 bc	0.19 ± 0.01 d	10.12 ± 0.80 bc	242.98 ± 4.37 a
Cd	14.65 ± 0.81 c	0.27 ± 0.01 b	10.15 ± 0.49 bc	226.34 ± 2.69 c
PEH1	14.51 ± 1.07 c	0.22 ± 0.01 cd	9.18 ± 0.08 cd	231.46 ± 0.73 b
PEH2	14.63 ± 0.46 c	0.25 ± 0.02 bc	8.62 ± 0.14 d	245.17 ± 1.23 a
*Factor* (*Df*)	7.17	9.21	12.14	127.1
*p* value	**	***	***	***

Note: Data are means ± SD (*n* = 3). Different letters indicate a significant difference in the results; the same letters indicate a non-significant difference in the results (*p*  <  0.05). CK: Control, PE1: 1.0% PE, PE2: 2.0% PE, PEH1: Cd + 1.0% PE, PEH2: Cd + 2.0% PE. **, *p* < 0.01; ***, *p* < 0.001.

**Table 2:** Cd concentrations and the total uptake of Cd in plant tissue and Cd concentration and bio-effectiveness in soil.

Treatments	Cd Concentration (mg kg^−1^)		Total Uptake (μg pot^−1^)		TF	Soil Total Cd Concentration	DTPA-Cd Concentration
	Shoot	Root	Shoot	Root		(mg kg^−1^)	(mg kg^−1^)
Cd	3.12 ± 0.27 c	45.74 ± 0.39 b	24.01 ± 2.46 b	36.26 ± 2.10 a	0.07 ± 0.01 b	3.16 ± 0.02 b	4.05 ± 0.02 b
PEH1	3.70 ± 0.09 b	49.66 ± 0.85 a	25.44 ± 1.80 b	36.77± 1.28 a	0.07 ± 0.01 b	3.53 ± 0.06 a	4.18 ± 0.05 a
PEH2	4.88 ± 0.20 a	50.80 ± 0.41 a	30.78 ± 1.20 a	32.73 ± 1.68 b	0.10 ± 0.01 a	3.24 ± 0.08 b	4.12 ± 0.07 ab
*Factor* (*Df*)	60.58	61.44	10.71	4.94	38.74	14.07	4.82
*p*	***	**	*	ns	***	**	ns

Note: Data are means ± SD (*n* = 3). Different letters indicate a significant difference in the results; the same letters indicate a non-significant difference in the results (*p* < 0.05). PEH1: Cd + 1.0% PE, PEH2: Cd + 2.0% PE. *, *p* < 0.05; **, *p* < 0.01; ***, *p* < 0.001, ns: There is no significant difference.

## Data Availability

The original contributions presented in this study are included in the article/Supplementary Materials. Further inquiries can be directed to the corresponding authors.

## Supplementary Materials

The following supporting information can be downloaded at: https://www.mdpi.com/article/10.3390/toxics13070555/s1, Figure S1: The combined effect of MPs and Cd on the antioxidant enzyme activity of plant shoot and root. (**A**), statistic data of SOD activity. (**B**), statistic data of POD activity; Figure S2: The linear model reveals the relative importance of soil environmental indicators (**A**) and plant physiological indicators (**B**) for the absorption of Cd in plants (%). Table S1: The combined effect of MPs and cadmium on the physicochemical properties of soil; Table S2: Significance levels (F values) of PE, Cd, and the impact of their interactions on measured variables according to two-way ANOVA analysis.
