# Supplementary material for: Coupled Effects of Polyethylene Microplastics and Cadmium on Soil–Plant Systems: Impact on Soil Properties and Cadmium Uptake in Lettuce
Source: Toxics. 2025 Jun 30;13(7):555. doi: 10.3390/toxics13070555 (PMC12299035; doi:10.3390/toxics13070555)
Supplement: Supplementary file 1 [file toxics-13-00555-s001.zip › toxics-3726838-supplementary.pdf]

# Coupled effects of polyethylene microplastics and cadmium on soil-plant systems: impact on soil properties response and plant cadmium uptake

Zhiqin Zhang [a, b *], Boyuan Bi [c*]

[a] School of Materials Engineering, Shanxi College of Technology, Shuozhou, Shanxi, 036000, China

[b] College of Natural Resources and Environment, Northwest A&F University, Yangling, Shaanxi, 712100, China

[c] Shaanxi Key Laboratory of Qinling Ecological Intelligent Monitoring and Protection, School of Ecology and Environment, Northwestern Polytechnical University, Xi' an, 710012, China

*** Corresponding author**

* Zhiqin Zhang (Tel: +86 18834910699, Email: zhangzhiqin@sxct.edu.cn)

* Boyuan Bi (Tel: +86 18700820263, Email: biboyuan0710@163.com)

**Figure captions:**

**Table S1** The combined effect of MPs and cadmium on the physicochemical properties of soil.

**Table S2** Significance levels (*F* values) of PE, Cd, and the impact of their interactions on measured variables according to two-way ANOVA analysis.

**Fig. S1** The combined effect of MPs and Cd on the antioxidant enzyme activity of plant shoot and root. A), statistic data of SOD activity. B), statistic data of POD activity.

**Fig. S2** The linear model reveals the relative importance of soil environmental indicators (A) and plant physiological indicators (B) for the absorption of Cd in plants (%).

**Table S1** The combined effect of MPs and cadmium on the physicochemical properties of soil.

| Trtment | Moisture (%) | pH | SOM ($g\ kg^{-1}$) | TN ($g\ kg^{-1}$) | TP ($g\ kg^{-1}$) |
|---|---|---|---|---|---|
| CK | 12.81 ± 1.98 a | 8.93 ± 0.02 a | 3.72 ± 0.16 c | 0.19 ± 0.01 c | 0.65 ± 0.01 ab |
| PE1 | 8.45 ± 0.63 b | 8.90 ± 0.03 b | 4.07 ± 0.16 b | 0.19 ± 0.01 c | 0.65 ± 0.02 ab |
| PE2 | 6.51 ± 0.30 c | 8.86 ± 0.04 c | 4.35 ± 0.03 a | 0.21 ± 0.01 a | 0.66 ± 0.01 a |
| Cd | 8.28 ± 1.56 b | 8.89 ± 0.01 b | 4.00 ± 0.06 b | 0.19 ± 0.01 c | 0.63 ± 0.01 b |
| PEH1 | 7.30 ± 0.35 bc | 8.86 ± 0.01 c | 4.10 ± 0.07 b | 0.20 ± 0.01 b | 0.64 ± 0.01 b |
| PEH2 | 7.14 ± 0.12 bc | 8.85 ± 0.01 c | 4.29 ± 0.26 a | 0.19 ± 0.01 bc | 0.63 ± 0.02 b |
| Factor (Df) | 26.74 | 14.96 | 13.97 | 7.69 | 3.26 |
| *P* value | *** | *** | *** | *** | * |

Note: Data are means ± SD. Different letters indicate a significant difference in the results; the same letters indicate a non-significant difference in the results ($P < 0.05$). CK: Control, PE1: 1.0% PE, PE2: 2.0% PE, PEH1: Cd + 1.0% PE, PEH2: Cd + 2.0% PE.

**Table S2** Significance levels (*F* values) of PE, Cd, and the impact of their interactions on measured variables according to two-way ANOVA analysis.

| Variables | *F* values and significant levels | | |
|---|---|---|---|
| | PE | Cd | PE × Cd |
| *Plant shoot* | | | |
| Fresh biomass | 18.22** | 30.52*** | 2.33ns |
| Dry biomass | 34.67* | 47.00*** | 0.21ns |
| Pn | 4.02* | 25.17*** | 1.32ns |
| Gs | 14.98*** | 0.01ns | 8.04** |
| Ci | 235.75*** | 105.5*** | 29.21*** |
| Tr | 12.37** | 35.93*** | 0.01ns |
| POD | 33.76*** | 66.12*** | 1.31ns |
| SOD | 8.69*** | 151.6*** | 6.57ns |
| $H_2O_2$ | 67.53*** | 225.8*** | 23.62*** |
| MDA | 21.76*** | 19.48*** | 8.79** |
| $O_2{\cdot}^-$ | 37.19*** | 133.2*** | 5.63* |
| Cd concentration | 60.58*** | 3459*** | 60.58*** |
| Cd uptake | 10.71** | 1805*** | 10.71** |
| *Plant root* | | | |
| Fresh biomass | 58.33*** | 36.41*** | 5.26* |
| Dry biomass | 27.71*** | 8.41* | 2.50ns |
| POD | 82.78*** | 125.3*** | 14.94*** |
| SOD | 39.89*** | 684.8*** | 20.59*** |
| $H_2O_2$ | 18.71*** | 24.56*** | 5.22* |
| MDA | 46.25*** | 179.1*** | 2.54ns |
| $O_2{\cdot}^-$ | 13.84*** | 89.16*** | 7.40** |
| Cd concentration | 61.44*** | 62066*** | 61.44*** |
| Cd uptake | 4.94* | 3794*** | 4.94* |
| TF | 38.74*** | 3473*** | 38.74*** |
| *Soil* | | | |
| Moisture | 38.03*** | 22.08*** | 17.78*** |
| pH | 25.01*** | 21.84*** | 1.47ns |
| SOM | 29.43*** | 2.58ns | 4.21* |
| TN | 9.20*** | 1.28ns | 9.39*** |
| TP | 0.27ns | 13.79*** | 1.00ns |
| Urease activity | 34.21*** | 44.39*** | 18.69*** |
| Alkaline phosphatase activity | 13.78*** | 1.30ns | 17.67*** |
| Catalase activity | 5.44* | 287.4*** | 0.96ns |
| Sucrase activity | 127.3*** | 3.86ns | 2.46ns |
| Soil total Cd concentration | 35.18*** | 30622*** | 35.18*** |
| DTPA-Cd concentration | 12.04*** | 153136*** | 12.04*** |

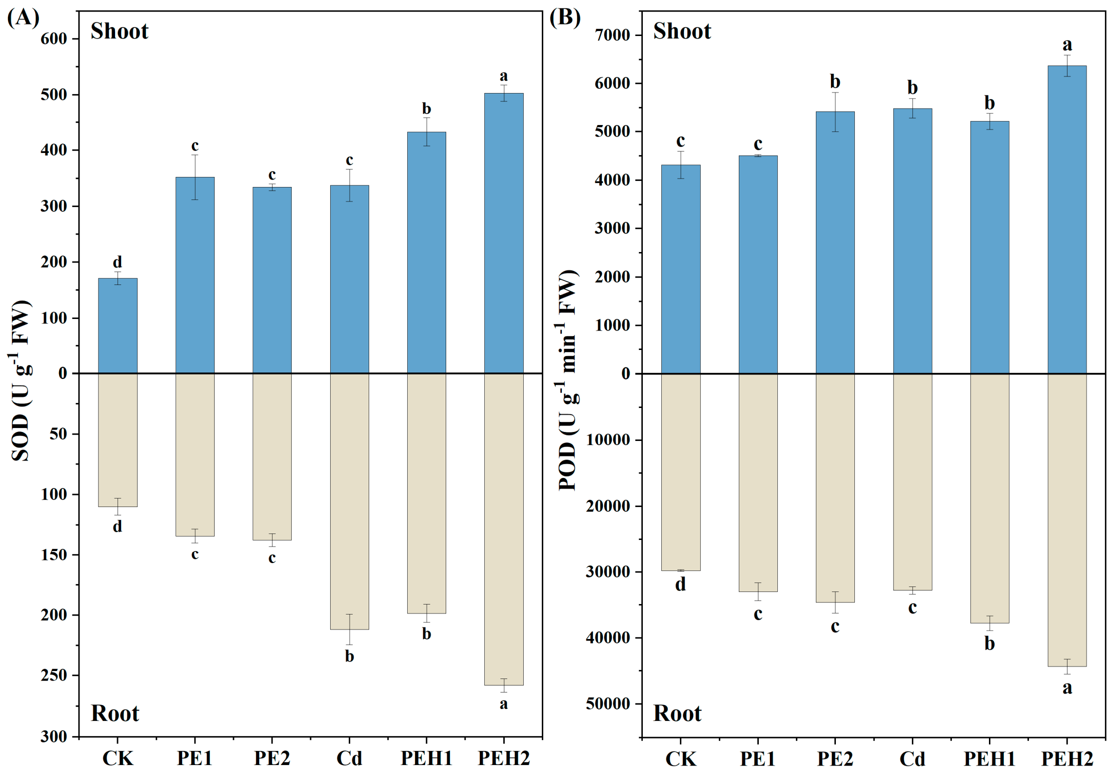


**Fig. S1** The combined effect of MPs and Cd on the antioxidant enzyme activity of plant shoot and root. A), statistic data of SOD activity. B), statistic data of POD activity. Note: Data are means ± SD. Different letters indicate a significant difference in the results; the same letters indicate a non-significant difference in the results ($P < 0.05$). CK: Control, PE1: 1.0% PE, PE2: 2.0% PE, PEH1: Cd + 1.0% PE, PEH2: Cd + 2.0% PE.

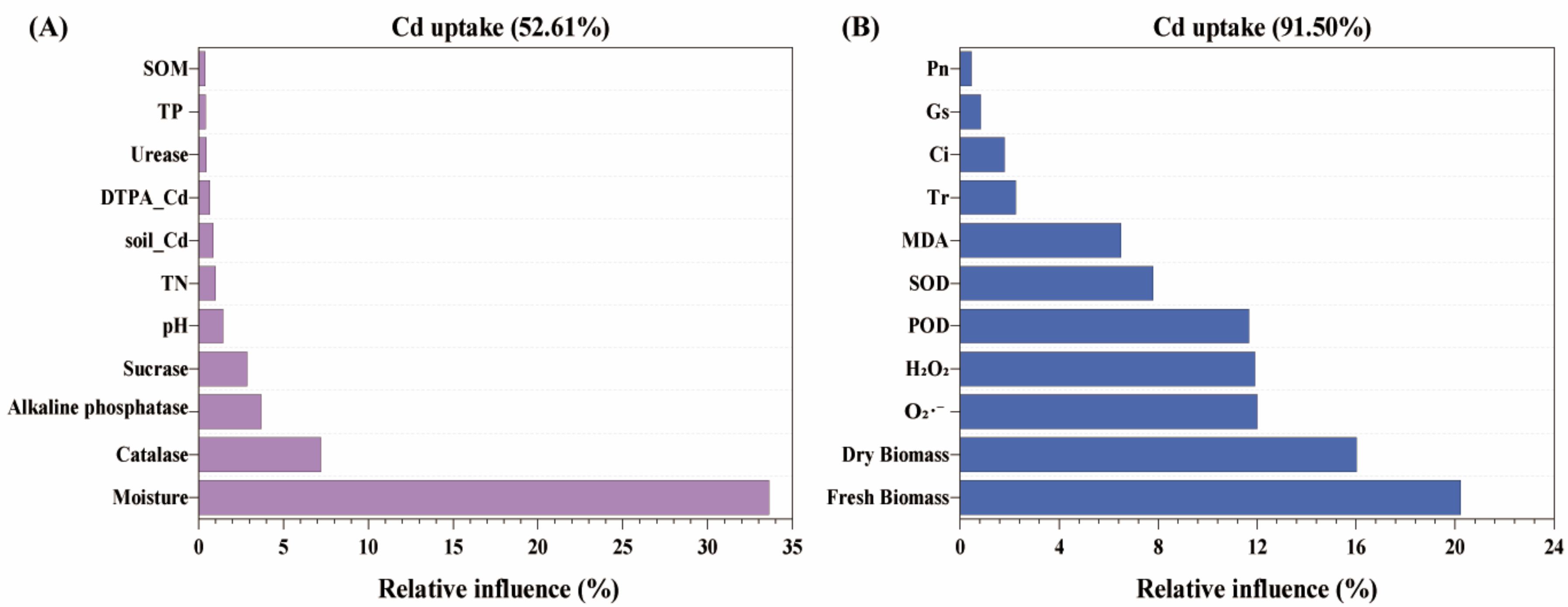


**Fig. S2** The linear model reveals the relative importance of soil environmental indicators (A) and plant physiological indicators (B) for the absorption of Cd in plants (%).
